# Detection of Autoantibodies against Recombinant Desmoglein 1 and 3 Molecules in Patients with Pemphigus vulgaris: Correlation with Disease Extent at the Time of Diagnosis and during Follow-Up

**DOI:** 10.1155/2009/187864

**Published:** 2009-12-10

**Authors:** Anna Belloni-Fortina, Diana Faggion, Barbara Pigozzi, Andrea Peserico, Matteo Bordignon, Vincenzo Baldo, Mauro Alaibac

**Affiliations:** ^1^Unit of Dermatology, University of Padua, Via Cesare Battisti 206, 35128 Padua, Italy; ^2^Veneto Institute of Oncology (IOV), IRCCS, Via Gattamelata 64, 35128 Padua, Italy; ^3^Department of Environmental Medicine and Public Health, Via Loredan 18, 35121 Padua, Italy

## Abstract

The recent availability of cDNA clones for pemphigus antigens has allowed the production of recombinant desmoglein 1 and desmoglein 3 molecules and the development of an ELISA approach in order to determine levels of antibodies to them. The aim of the study was to determine the relationship between autoantibodies levels and the extent of both mucosal and skin lesions in 20 patients with pemphigus vulgaris at the time of diagnosis and during follow-up. For the detection of autoantibodies by ELISA we used the recombinant proteins expressing overlapping sequences with the entire extracellular desmoglein 1 and desmoglein 3 domains. We showed that in presence of mucosal lesions there was a correlation between extension of mucosal involvement and autoantiboidies titres against both desmoglein 1 and desmoglein 3, whereas in presence of skin lesions there was a statistically significant correlation between extension of skin lesions and autoantibodies titres against desmoglein 3, but not against desmoglein 1. A not negligible number of patients showed variations of the desmoglein 3 autoantibodies titre which did not correlate with the severity of both cutaneous and mucosal involvement. Similar results were obtained analyzing autoantibodies titres against desmoglein 1. In conclusion, we believe that the utilization of recombinant desmoglein 1 and desmoglein 3 proteins by ELISA should be used with caution to monitor disease severity and response to therapy, although it remains a high specific test for the initial diagnosis of pemphigus and the identification of a change in the clinical phenotype of this condition.

## 1. Introduction

Pemphigus is a group of human autoimmune blistering disorders characterized by autoantibodies directed against transmembrane desmosomal proteins of keratinocytes called desmogleins (DSGs), resulting in loss of the normal epithelial cell-to-cell adhesion, through a process called acantholysis [1]. The two main subtypes of pemphigus are pemphigus vulgaris (PV) and pemphigus foliaceous (PF), and they have distinct clinical, histological, and immunopathological profiles [2, 3].

Direct immunofluorescence and serological indirect immunofluorescence have been for long time the main tests utilised to diagnose autoimmune blistering conditions. The recent availability of cDNA clones for pemphigus antigens has allowed the production of recombinant DSG1 and DSG3 molecules and the development of an enzyme-linked immunosorbent assay (ELISA) approach in order to determine levels of antibodies to them [4, 5]. This assay has shown a high sensitivity and specificity with respect to the possibility to diagnose pemphigus and to differentiate PV from PF. Patients with PV have circulating immunuglobulin G (IgG) against DSG3 and DSG1 [6], whereas PF patients only have anti-DSG1 IgG [7]. However, only few studies have correlated DSG1 and DSG3 autoantibodies levels and disease severity in PV.

The aim of the study was to determine the relationship between autoantibodies levels and the extent of both mucosal and skin lesions in PV at the time of diagnosis and during follow-up.

## 2. Material and Methods

The study comprised 20 caucasian patients with PV. The diagnosis was made on the basis of clinic, histologic, and immunopathologic criteria. We performed skin biopsies and collected serum from these subjects at the time of diagnosis and during follow-up. Hematoxylin-eosin stain and direct immunofluorescence were performed in each case.

For the detection of autoantibodies by ELISA we used the recombinant proteins expressing overlapping sequences with the entire extracellular DSG-1 and DSG-3 domains. These antigens have been provided (Medical & Biological Laboratories, Nagoya, Japan) as fusion proteins produced by baculovirus in “High Five insect cell line” (DSG1 e DSG3). Adding the serum of the patient to the recombinant proteins, we obtain an antigen-antibody reaction which determinates in turn a colorimetric reaction, whose adsorbance or optic density (OD) is noticed to 492 nm by an automatic spectrophotometry. ELISA was carried out using an ELISA-kit containing the relevant recombinant proteins (Medical & Biological Laboratories) and proved to be clinically reliable for the diagnosis of pemphigus vulgaris and foliaceus [8]. Positive controls for DSG1 and DSG3 were a diluted standard pemphigus foliaceus and pemphigus vulgaris serum, respectively. Negative control was diluted standard serum obtained from normal individuals. The cut-off values indicated by the manufacturer (autoantibody titre ≥20 Unit/mL) was used to discriminate positive from negative results. Disease extent was arbitrarily assessed as follows: no disease (−), mild disease (+) (less than 10% of skin involvement; 1 or 2 mucosal lesions and no difficulty in solid/liquid intake), moderate disease (++) (between 10 and 25% of skin involvement; 3–5 mucosal lesions and/or difficulty in solid intake), and severe disease (+++) (more than 25% of skin involvement; 6 or more mucosal lesions and/or difficulty in liquid intake). In presence of mucosal and/or skin lesions, Spearman's rank correlation test was used to correlate disease extent and autoantibodies titres. A *P* value of <.05 was considered significant. Analyses were performed using the EPI-Info (Centers for Disease Control of Atlanta, GA, USA).

## 3. Results

PV limited entirely to the mucosal surfaces was seen in both DSG3+/DSG1− patients and DSG3+/DSG1+ patients. Mucocutaneous involvement was observed in both DSG3+/DSG1− and DSG3+/DSG1+ patients, whereas PV limited entirely to the cutaneous surfaces was seen only in DSG3+/DSG1+. More precisely, a pattern of autoantibodies DSG3+/DSG1− was observed in 30% of patients, all showing a mucous or mucocutaneous involvement; a pattern DSG3+/DSG1+ was observed in 60% of patients, all with PV and involvement of the mucous membrane and/or skin. Two patients (one with mucous PV and one with mucocutaneous PV) showed a double negative DSG3−/DSG1− phenotype. In the 20 patients of the control group no one was positive for autoantibodies against DSG1, whereas one was positive for autoantibodies against DSG3. Autoantibodies patterns at the time of the diagnosis and IgG titres are summarized in Tables 1 and 2, respectively.

### 3.1. Autoantibodies Titres and Extent of Disease at the Time of Diagnosis

**Figure 1:**
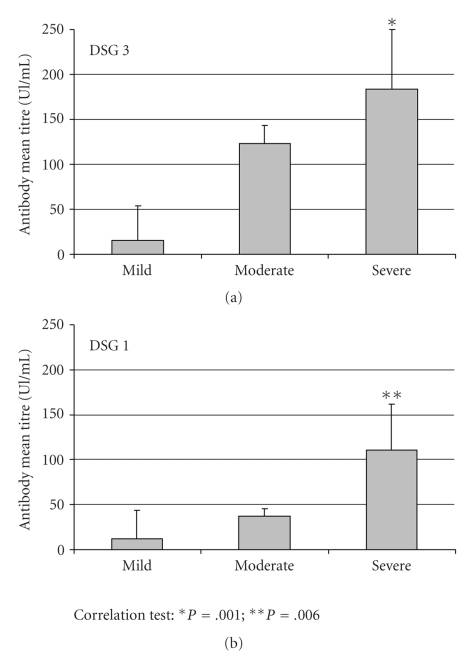
Anti-DSG3 and anti-DSG1 autoantibody mean titres in presence of mucosal involvement.

**Figure 2:**
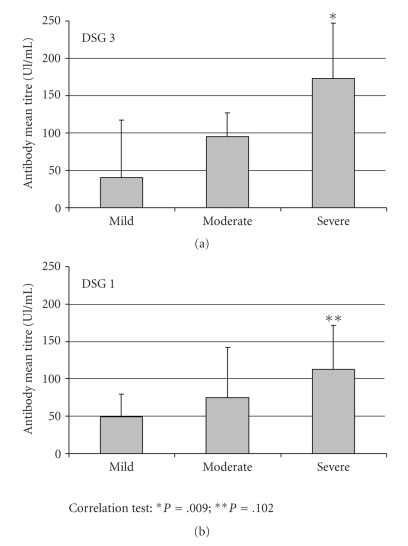
Anti-DSG3 and anti-DSG1 autoantibody mean titres in presence of skin involvement.

In presence of anti-DSG3 antibody titres above 20 Unit/mL, the mucosal involvement at the time of diagnosis was absent in 16,7% of patients, mild in 5,5% of patients, moderate in 50% of patients, and severe in 27,8% of patients. In patients with PV and positivity for the presence of anti-DSG3 autoantibodies, the skin involvement at the time of diagnosis was absent in 38,9% of patients, mild in 11,1% of patients, moderate in 22,2% of patients, and severe in 27,8% of patients. In patients with PV and positivity for the presence of anti-DSG1 autoantibodies, the mucosal involvement at the time of diagnosis was absent in 25% of patients, moderate in 33,3% of patients, and severe in 41,7% of patients. In patients with PV and positivity for the presence of anti-DSG1 autoantibodies, the skin involvement was absent in 25% of patients, mild in 8,3% of patients, moderate in 25% of patients, and severe in 41,7% of patients. In presence of mucosal lesions there was a correlation between extension of mucosal involvement and autoantibodies titres against both DSG-1 and DSG-3 (resp., *P* = .001 and *P* = .006), whereas in presence of skin lesions there was a correlation between extension of skin lesions and autoantibodies titres only against DSG-3 (*P* = .009), but not against DSG-1 (*P* = .102) (Figures 1 and 2).

### 3.2. Autoantibodies Titres and Extent of Disease during Follow-Up

13 patients repeated the test at least twice during follow-up. Results are summarized in Table 3. A not negligible number of patients showed variations of the DSG-3 autoantibodies titre which did not correlate with the extent of both cutaneous and mucosal involvement. Similar results were obtained analyzing autoantibodies titres against DSG-1 which unconstantly correlated with the extent of both mucosal and cutaneous disease.

In particular, there were some patients that continued to have high titers of autoantibodies against DSG-3, although a complete clinical remission in both cutaneous and mucosal compartments. It is interesting to note that we observed two patients with PV with only cutaneous involvement (VM and ZP) that showed a shift in autoantibodies profile from DSG3+DSG1+ to DSG3−DSG1+ which was clinically and histologically accompanied by a transition from PV to PF. This confirms the utility of this approach in the distinction of these two subgroups of pemphigus.

## 4. Discussion

The aim of our study was to establish the possible existence of a correlation between the extent of the disease at the time of diagnosis and the titre of autoantibodies against DSG1 and DSG3. Additionally, we investigated autoantibodies status during follow-up.

In our study, we observed a wide range of titres of autoantibodies against the recombinant protein DSG3; however the general tendency indicated that antibodies levels against DSG3 were more elevated in patients with moderate and severe skin and/or mucosal involvement if compared with patients with mild involvement. With regard to anti-DSG-1 antibodies we could demonstrate a statistically significant correlation between antibody serum levels and severity of mucosal involvement; there appears also to be a correlation between the severity of skin involvement and the level of DSG1 antibodies, although it was not statistically significant. Taken together, our findings are only partially in accordance with the study by Harman et al. [9] who found that levels of antibodies to DSG1 correlated with an increase in the severity of skin involvement, but not with the severity of mucosal involvement. The difference between the present investigation and the study by Harman et al. could be due to different criteria utilised for calculating the severity of disease. To this regard, it is possible that some aspects of disease severity (such as doses of medications required to control the disease) have not been captured by the simple evaluation of disease extent. Low power of sample size and racial differences between patients included in the studies [10] could also be responsible of these discrepancies indicating the need of additional large sample studies. On the other hand, the data presented in our study unequivocably show that there is a not negligible number of patients whose clinical presentation does not correlate with anti-DSG antibody titres. Consequently, therapeutic interpretation of this ELISA approach should be cautious.

From an immunopathogenetic point of view, the reasons for the presence of patients whose disease extent did not correlate with anti-DSG antibody titres, particularly anti DSG-1, are unclear. It is possible that antibodies against DSG1 recognize desmosomal antigens which are variable exposed in the epidermis in the initial phases of the condition, whereas the desmosomal antigen recognized by anti-DSG3 antibodies is expressed in the mucosa epithelium and skin at a more constant level.

We also investigated if patients with PV exhibited a correlation between the course of mucosal and skin lesions and variations of antibodies titre. We observed that there was no a preponderance of patients that showed a correlation between variation in the extent of the disease and the level of autoantibodies against DSG3 and DSG1. In particular, we observed some patients with significative ELISA values for autoantibodies against DSG3, who had responded to therapy and were in complete remission without mucosal and/or skin involvement. This is consistent with a recent investigation [11] and indicates that autoantibodies against the extracellular domains of this molecule are not always responsible of the disease; it is plausible that they may represent an epiphenomenon of an autoimmune reaction involving pathogenetic antibodies to nondesmoglein molecules or to the intracellular domain which are undetectable using our ELISA approach [12]. Characteristically, no positive values of DSG1 antibodies were observed in patients without clinical manifestations of the disease indicating that clinical remission coincides with a negative serology for these autoantibodies.

The autoantibodies against DSG-1 and DSG-3 may be induced in selected circumstances by a mechanism of epitope spreading which may explain those cases negative for these autoantibodies at the time of diagnosis (CR and AA see Table 1) which subsequently developed them. It remains also to be established the significance of their presence in patients with a complete clinical remission and absence of antigen exposition from lesional skin and/or mucosal tissue in relation to a future relapse of the disease.

Finally, in our investigation we have confirmed that this analysis is high specific to detect a change in the clinical phenotype of pemphigus. This was well evident in two patients where a serological shift from DSG3+/DSG1+ to DSG3−/DSG1+ was accompanied by a clinical transition from PV to PF. This is in line with our previous observation showing transition from PF to PV confirmed by a serologically shift from DSG3−DSG1+ to DSG3+DSG1+ [13]. It is interesting to note that in the present study this transition was observed in patients with PV with an exclusive cutaneous involvement.

In conclusion, we believe that the utilization of recombinant DSG1 and DSG3 proteins by ELISA should be used with caution to monitor disease severity and response to therapy, although it remains a high specific test for the initial diagnosis of pemphigus and the identification of a change in the clinical phenotype of this condition.

## Figures and Tables

**Table 1:** Autoantibodies patterns at the time of diagnosis.

	Exclusive skin involvement	Exclusive mucous involvement	Mucocutaneous involvement
DSG1+	15%	15%	30%
DSG3+			
DSG1−	/	20%	10%
DSG3+			
DSG 1−	/	5%	5%
DSG 3−			

**Table 2:** Autoantibodies titres at the time of diagnosis (Unit/mL).

Patient	DSG 1 titre	DSG 3 titre	Skin involvement	Mucosal involvement
A.A.	17,43	7,95	+	+
C.R.	16,8	1	—	+
C.G.	1	**30,66**	—	++
C.S.	**86,92**	**168,43**	++	++
D.A.	1	**23,51**	++	++
D.C.	1	**140,9**	—	++
F.S.	**140,97**	**250,17**	+++	+++
I.S.	**95,31**	**178,93**	+++	+++
L.M.C.	**67,46**	**210,66**	—	++
P.B.	**28,43**	**142,86**	—	++
P.E.	4,82	**72**	+	++
R.D.	**74,52**	**161.04**	—	+++
R.L.	**179,55**	**222,7**	+++	+++
R.S.	3,96	**169,69**	—	++
S.F.	**144**	**148,93**	++	++
T.E.	3,35	**38,34**	—	+
V.I.	**62,82**	**107,21**	+++	+++
V.M.	**85,19**	**106,25**	+++	—
V.L.	**66,46**	**39,38**	++	—
Z.P.	**126,74**	**41,08**	+	—

**Table 3:** Autoantibodies titres during follow-up. Values above the cutoff point of 20 Unit/mL are indicated in bold.

Name	Date	DSG 1	DSG 3	Skin	Mucosal
	16/12/03	17,43	7,95	+	+
A.A. (PV mucocutaneous)	24/04/04	**94.56**	**26,09**	+	+
	20/01/05	**60.9**	**21,92**	—	+
	22/03/07	16,80	1	—	+
C.R. (PV mucous)	12/04/07	**20,26**	**54,67**	—	+
	25/10/07	1	2,77	—	—
	20/12/06	1	**30,66**	—	++
C.G. (PV mucous)	06/06/07	0	**63,13**	—	—
	31/10/07	0	**122,82**	—	—
C.S. (PV mucocutaneous)	14/03/05	**86,92**	**168,43**	++	++
	18/10/06	**98,42**	**198,97**	++	—
F.S. (PV mucocutaneous)	04/09/03	**140,97**	**250,17**	+++	+++
	27/11/03	**25,76**	**189,19**	+	—
	02/03/04	0	**200,1**	—	—
	26/08/04	0	**34,28**	—	—
	20/12/04	0	**54,52**	—	—
	07/05/05	1	**90,22**	—	—
	05/09/05	0	**75,99**	—	—
	13/01/06	1,29	**85,7**	—	—
P.E. (PV mucocutaneous)	19/09/04	4,82	**72**	+	++
	01/09/05	1,4	**64**	—	+
	21/12/05	**74,52**	**161,04**	—	+++
R.D. (PV mucous)	07/03/06	19,06	**121,32**	—	++
	18/10/06	3	**179,78**	—	—
	03/12/03	**179,55**	**222,7**	+++	+++
	19/12/03	0	**140,06**	+++	+++
	19/01/04	**50,88**	**114,4**	++	++
	27/02/04	**38**	**143,9**	++	—
	03/06/04	**165,27**	**164,95**	++	—
R.L. (PV mucocutaneous)	14/03/05	**88,21**	**95,69**	+	—
	18/05/05	**135,38**	**131,07**	+	—
	20/06/06	**106,61**	**140,48**	+	—
	04/10/06	**148,44**	**159,12**	++	—
	24/01/07	**113,63**	**139,87**	+	—
	30/05/07	**124,23**	**154**	++	—
	09/03/05	3,96	**169,69**	—	++
R.S. (PV mucous)	23/05/07	0	**256,8**	—	++
	19/03/08	0	**175,69**	—	+
T.E. (PV mucous)	01/09/06	3,35	**38,34**	—	+
	28/02/07	1	**26,58**	—	—
	17/12/07	**62,82**	**107,21**	+++	+++
V.I. (PV mucocutaneous)	06/02/08	**56,73**	**193,26**	+++	++
	13/05/08	**27,59**	**179,46**	+	—
V.M. (PV cutaneous) Transition from pemphigus vulgaris to pemphigus foliaceous	26/10/06	**85,19**	**106,25**	+++	—
	23/05/07	**190**	0	++	—
	16/05/08	**144,6**	0	+	—
Z.P. (PV cutaneous) Transition from pemphigus vulgaris to pemphigus foliaceous	29/06/05	**126,74**	**41,08**	+	—
	09/11/05	**74,11**	10	+	—
	15/03/06	**115,53**	9,07	++	—
	26/07/06	**163,34**	17,78	++	—
	14/03/07	**80,48**	11,87	+	—

## References

[1] Sitaru C, Zillikens D (2005). Mechanisms of blister induction by autoantibodies. *Experimental Dermatology*.

[2] Yeh SW, Ahmed B, Sami N, Razzaque Ahmed A (2003). Blistering disorders: diagnosis and treatment. *Dermatologic Therapy*.

[3] Bystryn J-C, Rudolph JL (2005). Pemphigus. *The Lancet*.

[4] Lenz P, Amagai M, Volc-Platzer B, Stingl G, Kirnbauer R (1999). Desmoglein 3-ELISA: a pemphigus vulgaris-specific diagnostic tool. *Archives of Dermatology*.

[5] Amagai M, Komai A, Hashimoto T (1999). Usefulness of enzyme-linked immunosorbent assay using recombinant desmogleins 1 and 3 for serodiagnosis of pemphigus. *British Journal of Dermatology*.

[6] Sharma VK, Prasad HRY, Khandpur S, Kumar A (2006). Evaluation of desmoglein enzyme-linked immunosorbent assay (ELISA) in Indian patients with pemphigus vulgaris. *International Journal of Dermatology*.

[7] Amagai M, Tsunoda K, Zillikens D, Nagai T, Nishikawa T (1999). The clinical phenotype of pemphigus is defined by the anti-desmoglein autoantibody profile. *Journal of the American Academy of Dermatology*.

[8] Harman KE, Gratian MJ, Seed PT, Bhogal BS, Challacombe SJ, Black MM (2000). Diagnosis of pemphigus by ELISA: a critical evaluation of two ELISAs for the detection of antibodies to the major pemphigus antigens, desmoglein 1 and 3. *Clinical and Experimental Dermatology*.

[9] Harman KE, Seed PT, Gratian MJ, Bhogal BS, Challacombe SJ, Black MM (2001). The severity of cutaneous and oral pemphigus is related to desmoglein 1 and 3 antibody levels. *British Journal of Dermatology*.

[10] Harman KE, Gratian MJ, Bhogal BS, Challacombe SJ, Black MM (2000). A study of desmoglein 1 autoantibodies in pemphigus vulgaris: racial differences in frequency and the association with a more severe phenotype. *British Journal of Dermatology*.

[11] Abasq C, Mouquet H, Gilbert D (2009). ELISA testing of anti-desmoglein 1 and 3 antibodies in the management of pemphigus. *Archives of Dermatology*.

[12] Cheng SW, Kobayashi M, Tanikawa A, Kinoshita-Kuroda K, Amagai M, Nishikawa T (2002). Monitoring disease activity in pemphigus with enzyme-linked immunosorbent assay using recombinant desmogleins 1 and 3. *British Journal of Dermatology*.

[13] Pigozzi B, Peserico A, Schiesari L, Alaibac M (2008). Pemphigus foliaceus evolving into pemphigus vulgaris: a probable example of ‘intermolecular epitope spreading’ confirmed by enzyme-linked immunosorbent assay study. *Journal of the European Academy of Dermatology and Venereology*.

